# Design and Characterization of an “All-in-One” Lentiviral Vector System Combining Constitutive Anti-G_D2_ CAR Expression and Inducible Cytokines

**DOI:** 10.3390/cancers12020375

**Published:** 2020-02-06

**Authors:** Katharina Zimmermann, Johannes Kuehle, Anna Christina Dragon, Melanie Galla, Christina Kloth, Loreen Sophie Rudek, I. Erol Sandalcioglu, Belal Neyazi, Thomas Moritz, Johann Meyer, Claudia Rossig, Bianca Altvater, Britta Eiz-Vesper, Michael Alexander Morgan, Hinrich Abken, Axel Schambach

**Affiliations:** 1 Institute of Experimental Hematology, Hannover Medical School, 30625 Hannover, Germany; zimmermann.katharina@mh-hannover.de (K.Z.); galla.melanie@mh-hannover.de (M.G.); Kloth.Christina@mh-hannover.de (C.K.); Loreen.S.Rudek@stud.mh-hannover.de (L.S.R.); moritz.thomas@mh-hannover.de (T.M.); meyer.johann@mh-hannover.de (J.M.);; 2 Center for Molecular Medicine Cologne, University of Cologne, and Department I of Internal Medicine, University Hospital Cologne, 50931 Cologne, Germany; johannes.kuehle@uk-koeln.de; 3 Institute for Transfusion Medicine, Hannover Medical School, 30625 Hannover, Germany; Dragon.Anna@mh-hannover.de (A.C.D.); Eiz-Vesper.Britta@mh-hannover.de (B.E.-V.); 4 Department of Neurosurgery, Medical Faculty, Otto-von-Guericke University, 39120 Magdeburg, Germany; erol.sandalcioglu@med.ovgu.de (I.E.S.); belal.neyazi@med.ovgu.de (B.N.); 5 Department of Pediatric Hematology and Oncology, University Children’s Hospital Muenster, 48149 Muenster, Germany; rossig@ukmuenster.de (C.R.); bianca.altvater@ukmuenster.de (B.A.); 6 Regensburg Centre for Interventional Immunology (RCI), Department of Genetic Immunotherapy, and University Hospital Regensburg, 93053 Regensburg, Germany; Hinrich.Abken@klinik.uni-regensburg.de; 7 Division of Hematology/Oncology, Boston Children’s Hospital, Harvard Medical School, Boston, MA 02115, USA

**Keywords:** glioblastoma, “all-in-one” lentiviral vector, TRUCK, G_D2_CAR, NFAT, inducible cytokines, IL12, IL18

## Abstract

Genetically modified T cells expressing chimeric antigen receptors (CARs) so far have mostly failed in the treatment of solid tumors owing to a number of limitations, including an immunosuppressive tumor microenvironment and insufficient CAR T cell activation and persistence. Next-generation approaches using CAR T cells that secrete transgenic immunomodulatory cytokines upon CAR signaling, known as TRUCKs (“T cells redirected for universal cytokine-mediated killing”), are currently being explored. As TRUCKs were engineered by the transduction of T cells with two separate vectors, we developed a lentiviral modular “all-in-one” vector system that combines constitutive CAR expression and inducible nuclear factor of activated T cells (NFAT)-driven transgene expression for more efficient production of TRUCKs. Activation of the G_D2_-specific CAR via GD2^+^ target cells induced NFAT promoter-driven cytokine release in primary human T cells, and indicated a tight linkage of CAR-specific activation and transgene expression that was further improved by a modified NFATsyn promoter. As proof-of-concept, we showed that T cells containing the “all-in-one” vector system secrete the immunomodulatory cytokines interleukin (IL)12 or IL18 upon co-cultivation with primary human GD2^+^ tumor cells, resulting in enhanced effector cell properties and increased monocyte recruitment. This highlights the potential of our system to simplify application of TRUCK-modified T cells in solid tumor therapy.

## 1. Introduction

Adoptive cell therapy using chimeric antigen receptor (CAR)-modified T cells is one of the greatest breakthroughs in tumor therapy in recent years [1,2,3]. The authorization of Kymriah^®^ (Novartis) and Yescarta^®^ (Kite Gilead) underline CAR T cell efficacy and feasibility, especially of CD19-CAR T cells for the treatment of CD19^+^ B cell malignancies [4,5,6,7,8]. However, further improvements using CAR T cells are needed to address solid tumors. Heterogeneous antigen expression, insufficient tissue homing, and limited persistence of CAR T cells at the tumor site, as well as the immunosuppressive tumor microenvironment (TME), are major barriers that need to be overcome in the context of solid tumors [9,10,11,12]. Systemic application of interleukins, for example, interleukin (IL)12, provided some anti-tumor activity, as demonstrated by tumor regression and prolonged survival in murine studies [13,14], but was also linked with severe systemic toxicities [15,16].

In this direction, the TRUCK (“T cell redirected for universal cytokine-mediated killing”) concept was established, which equips CAR-redirected T cells with an additional NFAT (nuclear factor of activated T cells) inducible cytokine response that results in the restricted locoregional release of a transgenic cytokine upon CAR engagement of cognate target cells. The TRUCK approach can strengthen T cell activation and shape the TME by attracting innate immune cells to provide a more successful attack against tumor cells [17,18,19,20,21]. CAR T cells designed to release transgenic, inducible IL12 had improved cytolytic activity against solid tumors and increased the accumulation of activated macrophages and innate immune cells in the tumor tissue without inducing systemic toxicity in a murine model [19].

Of note, the TRUCK concept can also be transferred to other cytokines and transgenes of choice. IL18 was found to create a pro-inflammatory environment and increase T cell persistence in murine xenograft models [22]. In mouse models, IL18-secreting CAR T cells designed to target the carcinoembryonic antigen (CEA) showed superior anti-tumor activity against solid tumors in a pancreatic cancer model [18]. Additional studies indicated improved tumor control in leukemia and melanoma models using CD19-CAR T cells that constitutively express IL18 [23,24].

Current clinical trials underline the principle of CAR T cells with cytokine-enhanced activity. For example, CAR T cells engineered to secrete IL12 and target MUC16ecto-expressing solid tumors (NCT02498912), as well as CAR T cells targeting G_D2_ and secreting IL15 for cytokine support for the treatment of relapsed/refractory neuroblastoma, are being currently investigated in early phase trials (NCT03721068) (www.clinicaltrials.gov).

The generation of TRUCKs was originally accomplished by the transduction of two vector constructs, that is, one vector for constitutive CAR expression and one vector for inducible cytokine expression. The transduction of two vectors per T cell might enhance the risk of insertional mutagenesis by various genetic integrations and, thus, hinder manufacturing for clinical application. To overcome these limitations, T cell transduction protocols with single vectors were described, which mostly led to constitutive cytokine expression via an internal ribosomal entry site (IRES) or 2A site [14,23]. Regarding these aspects, we developed a modular “all-in-one” vector system that combines constitutive CAR expression and inducible CAR activation-induced cytokine secretion in a single vector. Additionally, the cytokine response is tightly linked to CAR expression and activation, so that cytokine expression would ideally be induced in the tumor upon recognition of the targeted solid tumor antigen. Thus, the “all-in-one” system should improve the safety and efficacy of T cell therapy, while avoiding systemic toxicity of anticancer principles.

As proof-of-concept, we generated a modular “all-in-one” vector system based on clinically applied third generation lentiviral self-inactivating (SIN) vectors designed to constitutively express a CAR in combination with NFAT-inducible cytokine expression, for example, IL12 or IL18. Therefore, we further codon-optimized a well-established second generation CAR that targets the cell-surface disialoganglioside G_D2_ [25], which has restricted expression in normal tissues, but is expressed on several solid tumors, including glioblastoma [26], neuroblastoma [27], and sarcomas [28]. Clinical trials already support the safety and efficacy of G_D2_-redirected CAR T cells without undesirable adverse effects [29,30,31]. However, response rates of neuroblastoma patients treated with G_D2_CAR T cells were lower than the successful treatment rates for CD19-CAR T cell therapies in leukemia patients [32], which reflects the need for improved therapies in solid tumor settings.

Here, we demonstrated the reliability of the established “all-in-one” vector construct regarding tight and effective induction of reporter genes and anti-cancer cytokines in primary human T cells that led to a potent anti-tumor effect in vitro, with the potential to reshape the TME in the case this system would be applied in vivo to treat solid tumors. The NFAT promoter was effectively and selectively induced after G_D2_CAR-specific activation by GD2^+^ target cells and patient-derived primary glioblastoma cells, which led to a strict CAR-activation-dependent cytokine secretion. Importantly, we showed that CAR T cell-secreted cytokines IL12 and IL18 led to increased recruitment of monocytes, as tested in a migration assay.

Thus, our “all-in-one” vector represents a useful tool for targeted applications to enhance the efficacy for future treatment of solid tumors.

## 2. Results

### 2.1. Specificity and Function of the G_D2_CAR

**Figure 1:**
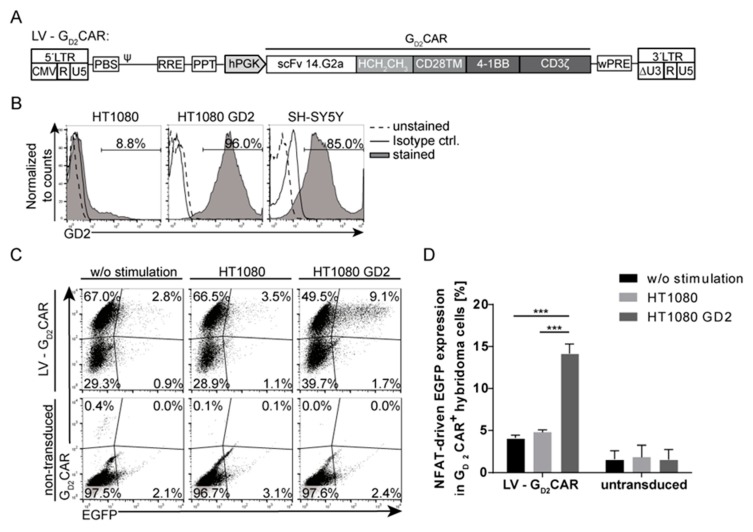
G_D2_ chimeric antigen receptor (G_D2_-CAR)-specific activation leads to nuclear factor of activated T cells (NFAT) promoter-driven enhanced green fluorescent protein (EGFP) upregulation. (**A**) Modular scheme of lentiviral self-inactivating (SIN) vector encoding a second generation G_D2_CAR expression cassette driven by the human phosphoglycerate kinase (hPGK) promoter. G_D2_CAR consists of the single chain variable fragment (scFv) 14.G2a, the HCH_2_CH_3_ hinge domain, the CD28 transmembrane domain (TM), and the 4-1BB/CD3ζ signaling domain. Depicted is the configuration after integration. Indicated are the long-terminal repeat (LTR) (ΔU3, R, and U5), primer binding site (PBS), packaging signal (ψ), rev-responsive element (RRE), central poly-purine tract (cPPT), and woodchuck hepatitis virus post-transcriptional regulatory element (wPRE). (**B**) Flow cytometric analysis of G_D2_-expressing target cell lines (HT1080 GD2 and neuroblastoma cell line SH-SY5Y) used for co-culture experiments. (**C**) Representative flow cytometric analysis of G_D2_CAR-transduced hybridoma cells after co-culture with indicated target cells. NFAT-driven EGFP upregulation was observed after co-culture with GD2^+^ target cells. Gated on G_D2_CAR^+^ hybridoma cells. (**D**) Summary of three independent co-culture experiments showing NFAT promoter-driven EGFP expression in G_D2_CAR-transduced hybridoma cells after stimulation with or without GD2^+^ target cells. Shown are mean values ± SD (*n* = 3). Indicated significance was determined by two-way analysis of variance (ANOVA) with Tukey’s multiple comparison test; *** *p* ≤ 0.001). CMV, cytomegalovirus.

We first reassessed the well-established second generation CAR targeting the cell-surface ganglioside G_D2_ with respect to specific target recognition. The G_D2_CAR contains a single-chain variable fragment (scFv) derived from the monoclonal antibody 14.G2a that has been approved for therapeutic use in children with neuroblastoma and has been used in previous clinical trials [33], an IgG1 (HCH_2_CH_3_) hinge domain followed by the CD28 transmembrane (TM) domain, and the intracellular 4-1BB and CD3ζ signaling domains [25] (Figure 1A). To verify the specificity and function of the G_D2_CAR, the CAR was initially cloned into a third generation lentiviral self-inactivating (SIN) vector [34] under the control of the human phosphoglycerate kinase (hPGK) promoter (LV-G_D2_CAR) and tested in a murine T cell hybridoma reporter cell line. The T cell hybridoma cells, which stably express a reporter construct consisting of an NFAT-sensitive minimal IL-2 promoter that induces enhanced green fluorescent protein (EGFP) expression upon CAR-specific activation, were transduced with the LV-G_D2_CAR vector construct [35] with a transduction efficiency of approximately 60%. NFAT-driven EGFP was upregulated after co-culture with HT1080 cells engineered to express the target antigen G_D2_ (~96%) (Figure 1B), while little EGFP upregulation was observed upon co-culture with wild-type HT1080 cells that mostly lack GD2 expression (~8%) (Figure 1C,D). Although only 15% of G_D2_CAR-transduced hybridoma cells upregulated EGFP upon G_D2_CAR-specific stimulation with HT1080 GD2 cells, this effect was sufficient to demonstrate the specificity of the G_D2_CAR for the GD2 antigen and that the CAR activation led to NFAT signaling in the modified T cells. Moreover, these results indicated that the CAR transmits an activation signal through NFAT that is entirely due to G_D2_CAR signaling, as the hybridoma cells do not express any endogenous human T cell receptor (TCR). These data provided the rationale for engineering the “all-in-one” vector based on the lentiviral G_D2_CAR vector.

### 2.2. Generation of “All-in-One“ Vector Constructs for Targeted Therapy

**Figure 2:**
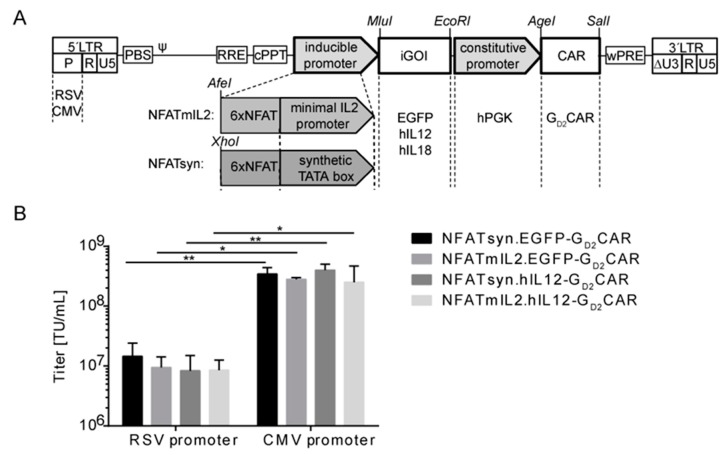
Schematic depiction of the modular “all-in-one” vector construct. (**A**) Lentiviral SIN vector scheme representing the main “all-in-one” construct elements and additional modifications. The vector plasmid configurations with indicated restriction sites are shown. The promoter sequence (P) in the 5’Long Terminal Repeat (LTR) is derived from Rous sarcoma virus (RSV) or cytomegalovirus (CMV). The construct is composed of a constitutively expressed G_D2_CAR element driven by a human phosphoglycerate kinase (hPGK) promoter and an inducible element consisting of six consensus NFAT enhancer repeats and the corresponding promoter (minimal IL2 promoter (mIL2) or synthetic TATA box (syn)) followed by the inducible gene of interest, respectively. (**B**) CMV-driven lentiviral “all-in-one” vector constructs led to improved lentiviral vector production. Constructs were pseudotyped with vesicular stomatitis virus glycoprotein (VSVg). Titration was performed in human HT1080 fibroblasts based upon G_D2_CAR expression as analyzed via flow cytometry three days post transduction. Bar graphs depict mean values ± SD (*n* = 3) of viral vector titers determined as described in Section 4.3. Indicated significance was determined by one-way ANOVA with Tukey’s multiple comparison test; ** *p* ≤ 0.01; * *p* ≤ 0.05; ns > 0.05). iGOI, inducible gene of interest.

To realize the TRUCK concept in a technically more applicable fashion, we combined inducible NFAT-driven cytokine secretion and constitutive CAR expression in a single vector and generated an “all-in-one” construct based on a clinically applied lentiviral SIN vector backbone (Figure 2A). To achieve inducible co-expression, an NFAT-driven cytokine cassette was inserted 3’ of the central polypurine tract (cPPT) followed by the G_D2_CAR expression cassette under control of the hPGK promoter, which enabled ubiquitous G_D2_CAR expression in nearly every transduced immune cell. For CAR-induced expression, we generated and tested two different NFAT-inducible promoters, namely NFATmIL2 and NFATsyn. Six NFAT consensus response elements (6xNFAT) were either fused to a minimal IL2 promoter to generate NFATmIL2 [36] or to a synthetic promoter element in addition to a synthetic TATA box to generate NFATsyn [37]. Both NFAT-responsive promoters were linked to an expression cassette for EGFP as an initial control module to optimize the vector configuration or the cytokine of interest. Each module of the vector was flanked by unique restriction sites to allow easy exchange of the respective modules. The designed architecture of the “all-in-one” vector constructs allowed independent secretion of the inducible gene of interest (iGOI), for example, EGFP control module, hIL12, or hIL18, driven by the CAR-activated NFATmIL2 or NFATsyn promoters. Compared with two-vector systems, direct linkage of CAR and iGOI within the “all-in-one” vector concept enables improved generation and control of genetically modified T cells and their inducible transgene expression.

Initial lentiviral particle productions using a vector configuration with the Rous sarcoma virus (RSV) promoter in the 5’Long Terminal Repeat (LTR) region resulted in low virus vector titers and required further improvement in order to generate titers relevant for clinical applications (Figure 2B). Thus, we generated and tested different vector configurations for improved production of viral vector particles by transduction and analyses of the human fibrosarcoma cell line HT1080. The exchange of the RSV promoter for the cytomegalovirus (CMV) promoter in the 5’LTR region of the SIN lentiviral vector plasmid resulted in a significant increase in lentiviral vector particle production to levels sufficient for clinical translation of “all-in-one” constructs (Figure 2B).

### 2.3. Promoter Choice of the Inducible Construct Determines the Extent of Activation

**Figure 3:**
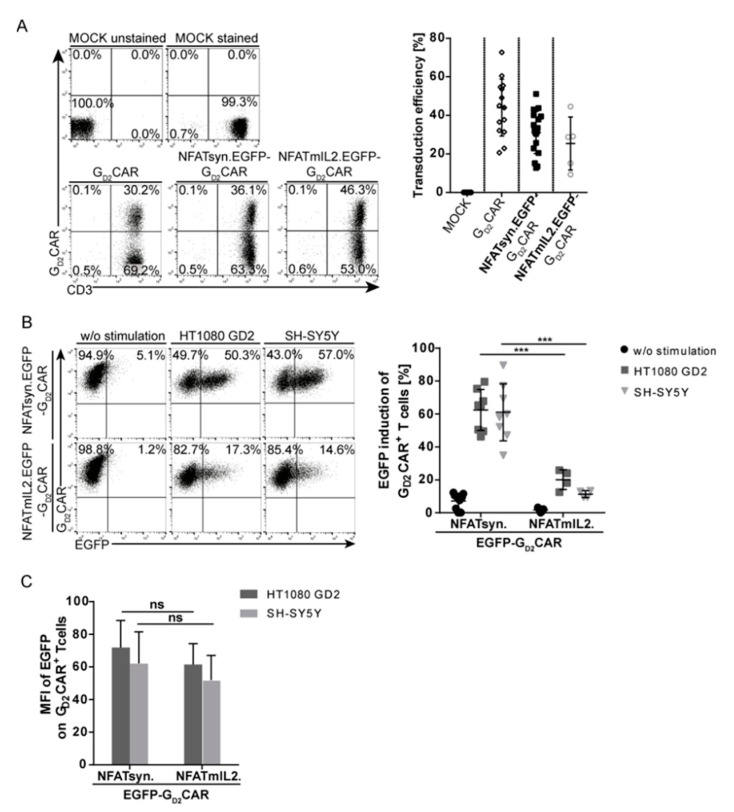
Promoter choice is critical for the amount of inducible transgene expression. (**A**) Representative flow cytometric analysis of primary human transduced CD3^+^ T cells. Transduction efficiencies were determined six days post transduction via a G_D2_CAR-specific antibody that recognizes the scFv region. The graph summarizes transduction efficiencies of the individual “all-in-one” constructs (NFATsyn.EGFP-G_D2_CAR or NFATmIL2.EGFP-G_D2_CAR) in primary human T cells (multiplicity of infection (MOI) 10). Shown are mean values ± SD (symbols indicate individual donors; *n* = 5–18). (**B**) Comparison of inducible promoters, namely NFATsyn and NFATmIL2. Primary human T cells were transduced with “all-in-one” EGFP constructs (NFATsyn.EGFP-G_D2_CAR or NFATmIL2.EGFP-G_D2_CAR), sorted, and co-cultured with GD2^+^ target cells. Both constructs drive NFAT promoter-regulated EGFP expression upon G_D2_CAR-specific activation. Representative flow cytometric analysis of NFAT-driven EGFP expression in transduced T cells after 24 h co-culture with a 6:1 effector/target (E/T) ratio. The graph summarizes multiple experiments of NFAT promoter-driven EGFP expression in “all-in-one” construct-positive (G_D2_CAR^+^) T cells post stimulation with GD2^+^ target cells. Shown are mean values ± SD; symbols indicate individual co-culture experiments with individual donors; *n* = 4–8. Indicated statistical significance was determined by one-way ANOVA with Tukey’s multiple comparison test; *** *p* ≤ 0.001. (**C**) Bars show mean fluorescent intensity (MFI) of inducible NFAT promoter-driven EGFP in “all-in-one” construct-positive (G_D2_CAR^+^) T cells after 24 h co-culture with GD2^+^ target cells from three independent experiments (E/T 6:1). Shown are mean values ± SD. Indicated statistical significance was determined by one-way ANOVA with Tukey’s multiple comparison test; ns > 0.05. MOCK: untransduced T cells.

To evaluate the potency and safety of the designed “all-in-one” vector constructs for adoptive T cell therapy, primary human T cells were transduced (vectors shown in Figure 2A), sorted, and tested for CAR-specific activation. Mechanistically, T cell activation via signaling domains of the constitutively expressed CAR leads to NFAT-mediated transcription, which results in the expression of the NFAT-responsive iGOI cassette. CAR surface expression in transduced T cells was confirmed by staining with a G_D2_CAR-specific antibody that recognizes the extracellular scFv domain. The application of viral vector particles at a multiplicity of infection (MOI) of 10 on T cells led to transduction rates of 30–50% for all tested constructs (Figure 3A). To test the proof-of-concept for the function of “all-in-one” vector constructs, we compared vectors harboring an EGFP expression cassette under control of two different NFAT-inducible promoters, NFATsyn and NFATmIL2. Marginal background EGFP expression was observed in unstimulated primary T cells expressing the “all-in-one” constructs (Figure 3B). Comparably moderate background EGFP expression was also observed in transduced primary T cells co-cultured with HT1080 (GD2-negative) target cells (Figure S1). In contrast, EGFP expression was highly induced in these T cells after G_D2_CAR-mediated activation upon co-culture with GD2^+^ target cells (HT1080 GD2 and SH-SY5Y) for 24 h (see Figure 1B for GD2 expression levels), and thus demonstrated the CAR-specific induction of the inducible expression cassette. Importantly, the NFATsyn promoter led to a ~3-fold higher EGFP expression compared with the NFATmIL2 promoter in transduced primary T cells. After stimulation with GD2^+^ target cells, the NFATsyn promoter led to a 60% EGFP upregulation in G_D2_CAR^+^ T cells compared with approximately 20% EGFP upregulation driven by the NFATmIL2 promoter (Figure 3B), with a similar density of EGFP expression as recorded by the mean fluorescent intensity (MFI) (Figure 3C).

The data indicate a tightly controlled EGFP expression upon G_D2_CAR-specific activation after recognition of its target antigen. Moreover, the choice of the promoter unit within the inducible cassette is a critical factor for the control of inducible transgene expression and leakiness. Owing to the higher induction capacity, the NFATsyn promoter-driven “all-in-one” construct variants were used in further analyses.

### 2.4. Induction of the NFATsyn Promoter after G_D2_CAR-Specific Activation with Patient-Derived Primary Glioblastoma Cells

**Figure 4:**
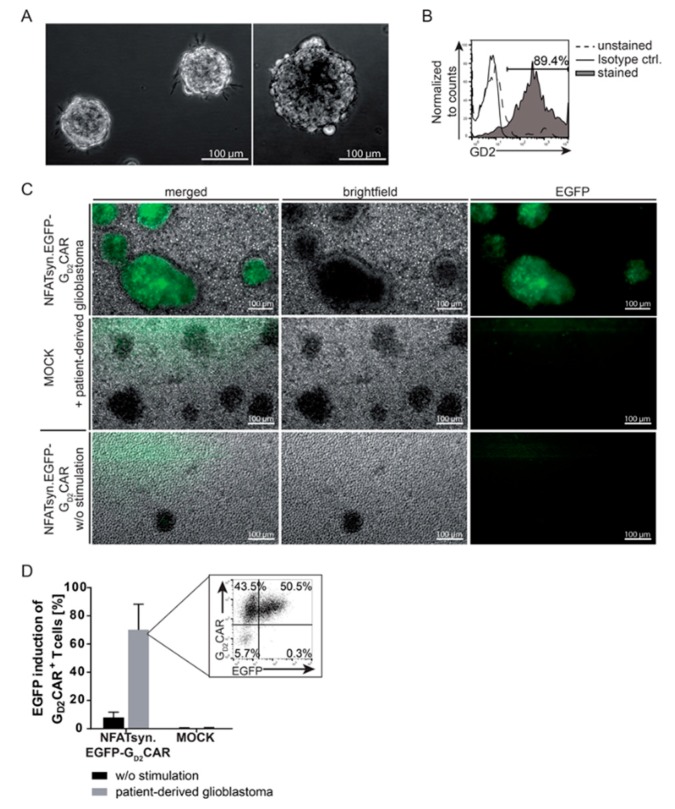
Specific induction of the NFATsyn promoter after CAR-specific activation with patient-derived primary glioblastoma cells. (**A**) Brightfield microscopy images of primary glioblastoma spheroids. (**B**) Expression of G_D2_ in primary glioblastoma cells analyzed via flow cytometric staining compared with isotype control (ctrl.) (representative patient sample). (**C–D**) Primary T cells from two independent donors were transduced with “all-in-one” NFATsyn.EGFP-G_D2_CAR construct and co-cultured with or without patient-derived glioblastoma cells for 24 h. MOCK represents non-transduced T cells. (**C**) Representative images of NFATsyn.EGFP-G_D2_CAR T cell co-culture experiments. The specific NFAT-driven EGFP upregulation after stimulation with patient-derived glioblastoma cells is shown in green. (**D**) Graph summarizes inducible NFAT-driven EGFP expression in “all-in-one” construct-positive (G_D2_CAR^+^) T cells post stimulation with GD2^+^ target cells and a representative flow cytometric analysis in transduced T cells after 24 h is depicted. Shown are mean values ± SD; symbols indicate individual co-culture experiments with individual donors; *n* = 2.

To further characterize the function of T cells modified with NFATsyn.EGFP.G_D2_CAR “all-in-one” vector constructs on primary target cells, we co-cultured “all-in-one” modified primary T cells with patient-derived GD2^+^ glioblastoma cells. After isolation, the patient-derived primary glioblastoma cells form typical spheroid structures (Figure 4A). Flow cytometric analysis revealed high expression of the GD2 antigen in these tumor cells in vitro (Figure 4B). Sorted primary T cells transduced with the NFATsyn.EGFP-G_D2_CAR and co-cultured with patient-derived glioblastoma cells specifically recognized the GD2-antigen expressed on glioblastoma. This became apparent by the NFAT-driven EGFP induction upon G_D2_CAR signal transduction, as shown by fluorescence microscopy (Figure 4C) and EGFP quantification by flow cytometry (Figure 4D). Compared with non-transduced T cells that did not show EGFP induction after cultivation with glioblastoma cells, NFATsyn.EGFP-G_D2_CAR transduced T cells specifically expressed EGFP and were enriched on or near the spheroid-forming glioblastoma cells that abundantly expressed the GD2-antigen (Figure 4C). Of note, the number of G_D2_CAR^+^/EGFP^+^ cells after co-culture with patient-derived glioblastoma cells (Figure 4D) correlated well with the EGFP upregulation after co-culture with HT1080 GD2^+^ and SH-SY5Y GD2^+^ cell lines (Figure 3B).

### 2.5. The Modular Design Facilitates Exchange of the Inducible Transgene and Allows Incorporation of the Promising Anticancer Cytokines IL12 and IL18

**Figure 5:**
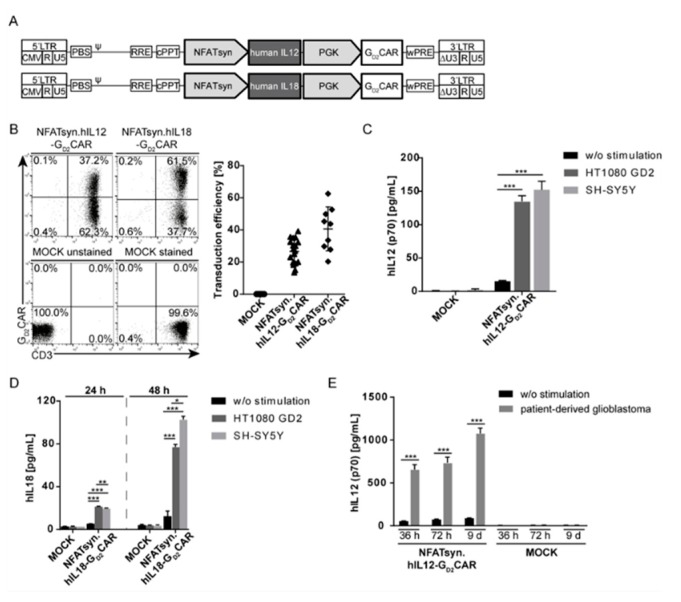
Effective human interleukin (IL)12 and human IL18 secretion after CAR-specific stimulation. (**A**) Schematic design of the “all-in-one” NFATsyn.hIL12-G_D2_CAR and NFATsyn.hIL18-G_D2_CAR constructs. Indicated is the inducible promoter (NFATsyn). (**B**) Representative flow cytometric analysis of primary human CD3^+^ T cells that were transduced with the “all-in-one” NFATsyn.hIL12-G_D2_CAR and NFATsyn.hIL18-G_D2_CAR constructs at an MOI of 10. Transduction efficiency was determined six days post transduction via a G_D2_CAR-specific antibody that recognizes the scFv region. The graph summarizes individual transduction efficiencies of the “all-in-one” NFATsyn.hIL12-G_D2_CAR and NFATsyn.hIL18-G_D2_CAR constructs in primary human T cells. Shown are mean values ± SD (symbols indicate individual donors; *n* = 9; MOI 10). (**C**) Human IL12 expression and (**D**) human IL18 expression in cell culture supernatants. Primary T cells were transduced with “all-in-one” NFATsyn.hIL12-G_D2_CAR and NFATsyn.hIL18-G_D2_CAR constructs and co-cultured with GD2^+^ target cells for 24 h (and 48 h). Inducible hIL12 or hIL18 secretion was determined via enzyme-linked immunosorbent assay (ELISA). ELISAs were performed in triplicates in two (hIL18) or three (hIL12) independent experiments; unsorted cells; 10:1 effector/target (E/T) ratio. Shown are mean values ± SD. Indicated significance was determined by one-way ANOVA with Tukey’s multiple comparison test; *** *p* ≤ 0.001; ** *p* ≤ 0.01; * *p* ≤ 0.05; ns > 0.05. (**E**) Human IL12 secretion after co-culture of primary T cells transduced with “all-in-one” NFATsyn.hIL12-G_D2_CAR with or without patient-derived glioblastoma cells for 36 h, 72 h, and 9 d. Inducible IL12 was determined via ELISA. ELISA was performed in duplicates. Shown are mean values ± SD. Indicated significance was determined by one-way ANOVA with Tukey’s multiple comparison test; (*** *p* ≤ 0.001).

As the modular design of the “all-in-one” vector construct also allows easy exchange of the iGOI, we next exchanged EGFP with cDNAs coding for human IL12 or IL18 (Figure 5A). Primary human T cells were transduced (MOI of 10) with the NFATsyn.hIL12-G_D2_CAR construct or the NFATsyn.hIL18-G_D2_CAR construct (Figure 5B) at similar efficiencies as the NFATsyn.EGFP-G_D2_CAR (see Figure 3A). Furthermore, G_D2_CAR-specific activation with GD2^+^ target cells induced NFAT-driven IL12 secretion after 24 h to levels of 150 pg/mL (Figure 5C) or IL18 (Figure 5D) secretion after 48 h to 100 pg/mL. In addition, unstimulated primary T cells expressing the “all-in-one” constructs as well as transduced primary T cells co-cultured with HT1080 (GD2-negative) cells induced marginal background human IL12 or human IL18 secretion (Figure S2), indicating a G_D2_CAR-specific induction of cytokines.

The higher induction capacity of the NFATsyn promoter compared with the NFATmIL2 promoter was also observed in co-culture experiments with the hIL12-G_D2_CAR (>100 pg/mL vs. <30 pg/mL) and hIL18-G_D2_CAR (30–80 pg/mL vs. <20 pg/mL) transduced T cells and G_D2_^+^ target cells (Figure S3).

To mimic a more clinically relevant setting, patient-derived glioblastoma cells were co-cultured with T cells transduced with the NFATsyn.hIL12-G_D2_CAR. Specific secretion of human IL12 after co-culture was observed and the amount of human IL12 in the cell culture supernatant increased over time to >1000 pg/mL (Figure 5E).

In conclusion, these results emphasize that the “all-in-one” vector constructs function properly in T cells co-incubated with primary patient-derived material.

### 2.6. “All-in-One” Vector Construct-Transduced Primary T Cells Exhibit a Superior Response with Regard to Cytotoxicity, Activation, and Immune Cell Recruitment

Primary human T cells transduced with the “all-in-one” constructs or the G_D2_CAR construct were analyzed for cytotoxicity against GD2^+^ target cells, T cell phenotype, and cytokine secretion profile.

**Figure 6:**
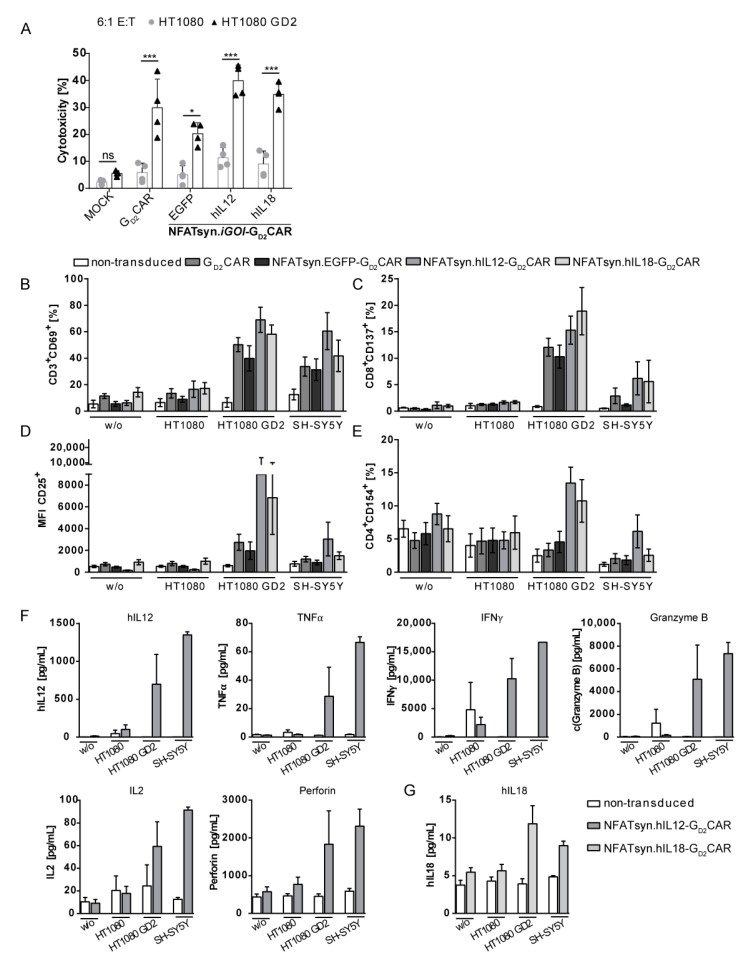
Cytotoxicity and activation marker analyses of “all-in-one” NFATsyn.iGOI-G_D2_CAR constructs-transduced primary T cells. (**A**) G_D2_CAR-specific signaling leads to target cell-specific killing. Lactate dehydrogenase (LDH) assay with supernatants of distinct “all-in-one” vector construct co-cultures. Cytotoxicity of sorted transduced primary human CD4^+^/CD8^+^ T cells (MOI 3) after 48 h co-culture with HT1080 and HT1080 GD2 target cells in a 1:6 effector/target (E/T) ratio. Shown are mean values ± SD (symbols indicate individual donors; *n* = 4). Indicated significance was determined by two-way ANOVA with Bonferroni’s multiple comparison test; *** *p* ≤ 0.001;; * *p* ≤ 0.05; ns > 0.05. (**B**–**E**) Flow cytometric analysis of T cell-specific activation marker of sorted “all-in-one” vector construct transduced primary human T cells after 48 h co-culture with indicated target cell lines in a 6:1 E/T ratio. (**B**) Percentage of CD3^+^CD69^+^ T cells; (**C**) percentage of CD8^+^CD137^+^ T cells; (**D**) mean fluorescent intensity (MFI) of CD25^+^ T cells; (**E**) percentage of CD4^+^CD154^+^ T cells after co-culture. Shown are mean values ±SD (symbols indicate individual donors; *n* = 5). (**F**) Cytokine analysis of co-culture supernatants harvested after G_D2_CAR-specific activation of human CD4^+^/CD8^+^ T cells transduced with “all-in-one” vector constructs. Cytokine concentrations were determined after 48 h co-culture experiments of transduced primary T cells stimulated with indicated target cells in a 6:1 E/T ratio using a customized LEDGENDplex™ Multi-Analyte Flow Assay (BioLegend), which allowed simultaneous detection of human IL12, tumor necrosis factor (TNF)α, Interferon (IFN)γ, granzyme B, IL2, and perforin. (**G**) Detection of human IL18. Shown are mean values ±SD (symbols indicate individual donors; *n* = 5).

To investigate whether the generated constructs enhance the cytotoxic properties of the T cells, we recorded the cytolytic activity of transduced (MOI of 3) and sorted T cells co-cultured with indicated target cells (Figure 6A). All T cells expressing a G_D2_CAR showed higher cytotoxicity against GD2^+^ target cells than non-transduced T cells, as measured by lactate dehydrogenase (LDH) release (Figure 6A). The similar level of in vitro cytotoxicity induced by the G_D2_CAR T cells regardless of cytokine expression/secretion capacity was expected, as these experimental conditions do not account for the potential beneficial recruitment of additional immune cells via inducible cytokine secretion.

We determined expression of T cell activation markers after co-culture with GD2^+^ target cells to investigate the G_D2_CAR-related activation of the engineered T cells. Here, we observed that co-culture with GD2^+^ target cells caused upregulation of CD3^+^CD69^+^ (Figure 6B) and CD8^+^CD137^+^ (Figure 6C) subpopulations in all T cells transduced with the constructs that contain a G_D2_CAR. Moreover, T cells transduced with the NFATsyn.hIL12-G_D2_CAR and NFATsyn.hIL18-G_D2_CAR had increased expression levels of CD25 (based on MFI) upon co-culture with HT1080 GD2 cells (Figure 6D). In contrast, CD154 expression on CD4^+^ T cells was expressed at similar levels in all analyzed T cell groups, except for a slight increase of CD4^+^CD154^+^ NFATsyn.hIL12-G_D2_CAR and NFATsyn.hIL18-G_D2_CAR T cells after co-culture with HT1080 GD2 cells (Figure 6E). In accordance with the cytotoxicity analyses, G_D2_CAR containing T cells had upregulated expression of the analyzed activation markers, which underlines the specific activation potential of the transduced T cells compared with non-transduced T cells.

In addition to the activation markers, we also analyzed the expression levels of pro-inflammatory cytokines in supernatants from co-cultures of “all-in-one” vector transduced T cells in response to indicated target cells. Human primary T cells transduced with the NFATsyn.hIL12-G_D2_CAR “all-in-one” construct showed increased hIL12 cytokine levels upon co-culture with GD2^+^ target cells and upregulation of pro-inflammatory cytokines like tumor necrosis factor (TNF)α, Interferon (IFN)γ, and IL2, as well as cytolytic effector cytokines like perforin and granzyme B (Figure 6F). These results indicated a pro-inflammatory potential compared with non-transduced or other transduced T cells, as indicated (Figure S4). We also observed an increase in hIL18 secretion by NFATsyn.hIL18-G_D2_CAR T cells upon co-culture with GD2^+^ target cells (Figure 6G).

**Figure 7:**
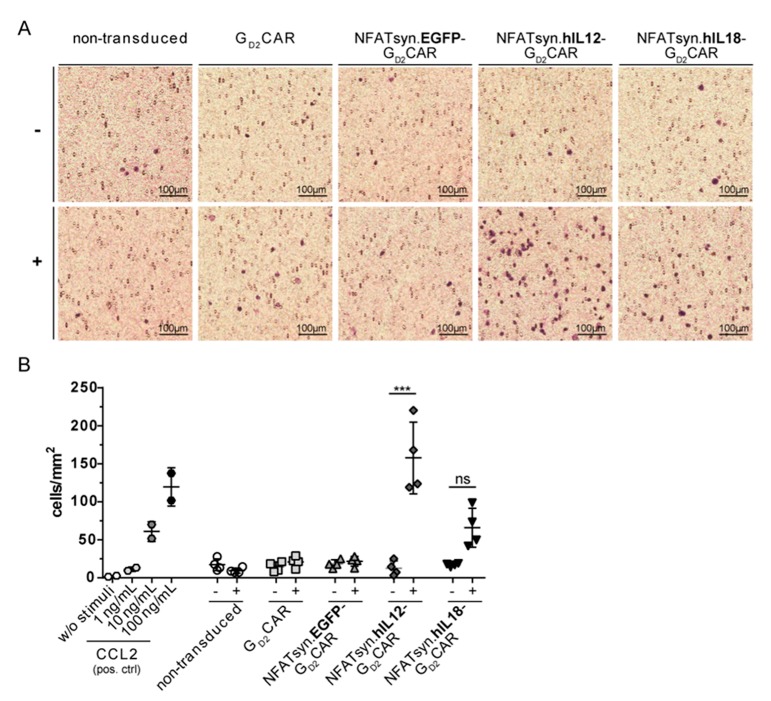
“All-in-one” NFATsyn.IL12-G_D2_CAR and NFATsyn.IL18-G_D2_CAR vector constructs exhibit higher recruitment potential of monocyte cell line MONO-MAC-6 (MM6) shown in a cell migration assay. Cell culture supernatants from co-culture experiments performed with “all-in-one” vector/G_D2_CAR constructs transduced primary T cells (sorted) and GD2^+^ target cells (E/T 6:1) were collected after 48 h and used in a cell migration assay with modified Boyden chambers and 5 × 10^5^ MM6 cells/well. Supernatants from cells w/o stimulation served as controls. **(A)** Membranes were fixed and Giemsa stained after 4 h incubation. (**B**) Graph summarizes four independent experiments (each performed in duplicates) of migrated cells influenced by the different cell culture supernatants of the “all-in-one” constructs harvested after co-culture with (+) or without (−) HT1080 GD2^+^ target cells. Different concentrations of C-C motifchemokine ligand 2 (CCL2) were used as positive control. Shown are mean values ± SEM. Indicated significance was determined by one-way ANOVA with Tukey’s multiple comparison test; *** *p* ≤ 0.001; ns > 0.05.

To demonstrate the biological activity of G_D2_CAR-induced cytokines with respect to the recruitment of additional immune cells, we used a modified Boyden chamber assay to compare the migration potential of cell supernatants collected from co-culture experiments of transduced primary T cells and GD2^+^ target cells (HT1080 GD2) to promote the recruitment of monocytes. The capacity of IL12 and IL18 containing supernatants to recruit the monocytic cell line MONO-MAC-6 (MM6) was shown by positive Giemsa staining of the transwell membrane through which migrating cells were recruited (Figure 7A). Giemsa staining of migrated monocytes revealed that greater numbers of MM6 cells were recruited by supernatants collected from “all-in-one” NFATsyn.hIL12-G_D2_CAR and NFATsyn.hIL18-G_D2_CAR transduced T cells co-cultured with HT1080 GD2 target cells as a result of induced cytokine secretion (IL12 and IL18). Moreover, supernatants from NFATsyn.hIL12-G_D2_CAR transduced T cells showed a statistically significant effect for improved monocyte recruitment. Supernatants from NFATsyn.hIL18-G_D2_CAR transduced T cells (Figure 7B) indicated a trend for a better recruitment potential. Taken together, supernatants from T cell subsets expressing “all-in-one” constructs designed to deliver cytokines in an inducible fashion upon CAR activation by antigen recognition exhibited an enhanced immune cell recruitment potential in vitro.

## 3. Discussion

We describe a method to generate TRUCKs using an “all-in-one” vector system for targeted adoptive cell therapy that combines constitutive CAR expression and inducible cytokine secretion. The system is therapeutically relevant, especially with regard to the treatment of solid tumors, as it redirects T cells to the target cells through the scFv of the constitutively expressed CAR and allows NFAT-driven cytokine induction only upon tumor target cell binding. This allows TRUCK T cells to shape the local tumor microenvironment and create a pro-inflammatory milieu by recruiting additional innate immune cells to help further eliminate the tumor. Previously published concepts of CAR T cells that secrete additional effector molecules are mostly based on transduction protocols that involve the delivery of two-vectors [19,38]. Recently, Liu et al. described a one-vector system for inducible cytokine expression driven by an NFAT promoter element with six NFAT response elements that is activated following activation of a constitutively expressed Glypican-3 (GP3)-CAR, and they nicely demonstrated the functionality of their one-vector system in in vivo mouse models for hepatocellular carcinoma (HCC) [21]. Similar to the one-vector system by Liu et al., the vector system described here offers a versatile cell modification strategy. In contrast to Liu et al., we used a different vector configuration for our “all-in-one” approach and basically focused on the establishment of an “all-in-one” vector system to further develop and improve the TRUCK concept. Moreover, the modular one-vector design that we describe here allows the easy exchange of the different cassettes, and thus facilitates adaptation for application in various settings. Transduction efficiencies of about 30–60% in primary human T cells were achieved with low MOIs. This was obtained after the introduction of a CMV promoter into the 5’LTR region of lentiviral transfer plasmids. This modification enabled the production of lentiviral “all-in-one” constructs with clinically applicable titers, with viral vector titers that were up to 15-fold higher than those achieved with previous vector systems containing the RSV promoter. This advance facilitates the production of high numbers of gene-modified T cells as the basis for further adoptive CAR T cell therapies. In contrast to previous procedures, only a single vector integration event is needed with this one-vector system, which also reduces the risk of insertional mutagenesis that was described as a potential side effect of retroviral gene transfer [39]. Although no severe adverse events have been reported in the context of lentiviral gene therapy studies in hematopoietic stem cells or human primary T cells, these studies did not test the safety of cells modified with more than one vector [40,41]. Furthermore, dual transductions also require two viral vectors produced under good manufacturing practices (GMPs), which is cost-intensive and laborious. Therefore, our developed “all-in-one” vector system simplifies the application of TRUCK modified T cells for tumor therapy and simplifies translation of this therapeutic concept for use by the scientific community. The tight linkage of CAR activation and cytokine secretion was also demonstrated for the “all-in-one” vector, as the induction of the desired protein of interest only occurred after G_D2_CAR-specific activation, for example, by co-cultivation of the “all-in-one” transduced CAR T cells with GD2^+^ target cells, which resulted in NFAT promoter-driven cytokine induction and secretion. This is not the case for CAR T cells that constitutively express an immune modulatory cytokine via an IRES or 2A site.

Stably integrated reporter cell lines for transgene expression harboring activation-inducible promoters were already described for functional testing of T or natural killer (NK) cell activation, as well as in the context of CAR T and NK cell therapy [42,43]. These reporters are mostly based on NFAT-, nuclear factor kappa-light-chain-enhancer of activated B cells (NFkB)-, and activator protein (AP)-1-based promoters, as activation pathways in T and NK cells are mediated by these transcription factors. Several studies used reporter constructs driven by NFAT response elements to analyze activation and signal transduction pathways in primary T cells [43,44]. In this regard, synthetic NFAT promoters were previously used for inducible transgene expression [13,19], but mainly in a separate vector that was transduced in addition to the CAR vector system. Here, we compared two different inducible NFAT-based promoters fused to the transgene of interest within the lentiviral “all-in-one” vector system in primary human T cells to improve the TRUCK concept. These promoters both included six NFAT response consensus enhancer elements, but differed in their fused core promoters and TATA boxes, namely the minimal IL2 promoter and the synthetically designed core promoter plus TATA box, which was limited to sequences essential for expression [36,37]. Although both promoters were able to specifically induce transgene expression after CAR-specific activation, the NFATsyn promoter more potently induced transgene expression with significantly higher levels of EGFP, hIL12, or hIL18 production after CAR-specific activation in primary human T cells. These data indicated that, in addition to the number of response elements, the choice of the fused promoter is also of major importance for high-level inducible transgene expression. Moreover, the percentage of modified cells and the transgene expression levels could be titrated to the needed extent by modulation of the promoter. However, cell-type dependency and the feasibility of tight induction would need to be evaluated for further use of “all-in-one” vectors in other therapeutic cell types.

The pro-inflammatory cytokines IL12 and IL18 were studied in the context of our “all-in-one” vector system as they are promising therapeutic candidates for the treatment of solid tumors. IL12 has been explored to treat several malignancies in preclinical models and clinical applications, including melanoma and ovarian cancer. However, several cytokine-related toxicities were described and represent one of the main limitations of systemic delivery or constitutive expression via retroviral vectors in tumor therapy [15,45,46]. The “all-in-one” vector system represents a potential solution to overcome these toxicities as the inducible cytokine secretion is tightly linked to the activation of the CAR T cell, and thus ensures local and temporal cytokine expression only at the tumor site. One important aspect that needs to be addressed, but is difficult to evaluate, is a maximal baseline-expression level of the applied cytokines to allow local therapeutic effects without systemic side effects. We observed that the NFATsyn promoter provides a combination of high induction and low-baseline expression levels. However, most CAR T cell-targeted antigens expressed on solid tumors are not exclusively expressed on tumors, but are also present in normal tissues, which might result in unwanted “on target/off tumor” toxicities [10]. Thus, low-baseline expression without CAR signaling is clearly of benefit in this respect.

The G_D2_CAR-specific activation after stimulation with GD2^+^ target cells resulted in the upregulation of T cell-specific activation markers, without the additional increase of activation markers observed in T cells containing an inducible cytokine cassette. The effect of the induced IL12 cytokine expression in target antigen-activated NFATsyn.hIL12-G_D2_CAR transduced T cells was also indicated by increased expression levels of several pro-inflammatory cytokines released by the engineered T cells. This underlines the potential benefit of the designed construct to recruit additional immune cells. The described benefit of the additional cytokine secretion by T cells was also observed in the in vitro cell migration assays with MM6 cells. The resulting cytokine release recruited monocytes, and thus provided proof-of-concept that our system could contribute to the creation of a pro-inflammatory environment in the treatment of solid tumors. These promising in vitro results should be confirmed in appropriate in vivo models to further address the pro-inflammatory potential of the anticancer cytokines to combat solid tumors. Thus, T cells transduced with “all-in-one” constructs should be tested in a humanized, clinically relevant mouse model, to determine their in vivo potency regarding attraction and recruitment of additional immune cells and long-term cytotoxic effects of the secreted cytokines.

Another potential application for the “all-in-one” constructs is to screen CARs and TCRs in reporter assays [42,43,44] by displaying CAR libraries, including different scFvs, hinge regions, and signaling domains directly in primary human immune cells. The CAR function can be directly linked to the NFAT promoter-driven iGOI expression or cytokine secretion and can be investigated after target antigen recognition. This allows the investigation of CAR specificity, functionality, efficacy, and dose-dependent cytokine secretion studies in one molecule.

The described vector system combines the advantage to exchange different modules of the construct to facilitate manufacturing for clinical application and reduce the risk of adverse events like insertional mutagenesis. Thus, the NFAT-promoter, as well as the inducible gene of interest, could be easily replaced by other promoters, reporters, and cytokines to adapt the “all-in-one” vector construct to other immune cell-based adoptive therapies. Adaptation to tumor therapies using checkpoint inhibitors is also conceivable [47,48]. Alternatively, the “all-in-one” construct technology can be used for the application of anti-inflammatory therapies, for example, autoimmune diseases or transplantation studies. In this regard, this system could be applied to genetically manipulate regulatory T cells to express anti-inflammatory cytokines, like IL10 or TGF-β [49]. Hence, the “all-in-one” system offers a useful technology for various future therapeutic purposes, especially with regard to improved viral vector design and viral vector particle generation, and is transferable to other immune cells, vector systems, and cytokines.

## 4. Materials and Methods

### 4.1. Human Material

All experiments were performed with residual blood samples from platelet (PLT) apheresis disposables used for routine PLT collection and from regular anonymous healthy donors. As a requirement for donations, the respective donors had no signs of acute infection or a previous history of blood transfusion. The studies were approved by the local ethics committee (approval number 2519-2014).

### 4.2. Cell Lines 

293T cells (#ACC 635; DSMZ, Brunswick, Germany) were used for lentiviral production and cultured in Dulbecco’s modified Eagle’s medium (DMEM) (Biochrom, Berlin, Germany) supplemented with 10% heat-inactivated fetal bovine serum (FBS), 100 U/mL penicillin, 100 μg/mL streptomycin, and 1 mM sodium pyruvate (all PAN Biotech, Aidenbach, Germany). In functionality and cytotoxicity experiments, the fibrosarcoma cell line HT1080 (#ACC 315; DSMZ), the HT1080 equipped with GD2 by retroviral transduction of a GD3S/GD2S transgene, and the established neuroblastoma cell line SH-SY5Y were used. All cell lines were cultivated similarly in DMEM. Primary glioblastoma samples were obtained from glioblastoma tumor resections after receiving the patient’s informed consent (Nordstadt Cerebral Cancer Study (NoCCa-Study) Register- Nr. 6864). Cells were isolated and cultivated until further usage according to Hasselbach et al. [50] with modifications. In brief, the isolated spheroids were cultivated in DMEM/F12 (Gibco, Karlsruhe, Germany) supplemented with 100 U/mL penicillin, 100 μg/mL streptomycin, N2 supplement (1×) (Miltenyi Biotec B.V. & Co. KG, Bergisch Gladbach, Germany), 0.5 mg/mL bovine serum albumin (BSA) (PAN Biotech), 25 µg/mL gentamicin (Gibco), 20 ng/mL human basic fibroblast growth factor (bFGF), and human epidermal growth factor (EGF) (both PeproTech, Hamburg, Germany) in low-attachment flasks (Corning, Wiesbaden, Germany) until further usage. Primary T cell cultures and T cell-related assays were performed in Roswell Park Memorial Institute (RPMI) 1640 (PAN Biotech), supplemented with 10% FBS (PAN Biotech), 100 U/mL penicillin, 100 μg/mL streptomycin, 0.05 mM β-mercaptoethanol (Sigma-Aldrich, Darmstadt, Germany), 20 mM 4-(2-hydroxyethyl)-1-piperazineethanesulfonic acid (HEPES) (PAN Biotech), 1% Minimal Essential Medium (MEM) non-essential amino acid solution (Gibco), 1% sodium pyruvate (PAN Biotech), and 100 lU/mL human IL-2 (ProLeukin S, Clinigen Healthcare B.V.). For some experiments (Figure 6, Figure 7 and Figure S4), T cell assays were performed in Tex magnetic activated cell sorting (TexMACS) medium (Miltenyi) supplemented with 3% human AB serum (c.c.pro, Oberdorla, Germany), and 12.5 ng/mL human IL-7 and human IL-15 (PeproTech, Rocky Hill, CT, USA) in humidified incubators at 37 °C and 5% CO_2_.

### 4.3. Cloning of “All-in-One” Vector Constructs, Production, and Titration of Viral Supernatants

For the generation of NFATsyn.EGFP.GD2-CAR (pRRL.PPT.NFATenh.synTATA.EGFP. PGK.newMCS.GD2CAR.PRE), multiple cloning steps were performed. In brief, the synthesized NFATenh.synTATA.EGFP expression cassette (Thermo Fisher Scientific, Regensburg, Germany) was designed according to Merlet et al. [37], slightly modified, and individual modules (NFATenhancer, synTATA, and EGFP) were equipped with unique restriction sites (Figure 2A). After verification of the sequence by Sanger sequencing, NFATenh.synTATA.EGFP was removed from the shuttle vector via XhoI and EcoRI and introduced into pRRL.PPT.PGK.newMCS.EBFP2.i2.Zeo.PRE via a three-fragment ligation using 529 bp *EcoR*I/*Age*I and 7871 bp *Age*I/*Xho*I fragments of pRRL.PPT.PGK.newMCS.EBFP2.i2.Zeo.pre. Subsequently, the EBFP2.i2.Zeo cassette of the resulting pRRL.PPT.NFATenh.synTATA [37]. EGFP.PGK.EBFP2.i2.Zeo.PRE plasmid was replaced by AgeI and SalI with the open reading frame (ORF) of the G_D2_-CAR that was optimized for human codon-usage. For the generation of the NFATmIL2.EGFP.GD2-CAR, the 6xNFAT-mIL2 promoter plus EGFP cDNA was amplified from previously published construct SIN-(NFAT)_6_-GFP [36] via PCR with 5’ flanking *Sal*I + *Afe*I restriction sites and 3’ flanking *EcoR*I + *Nhe*I restriction sites. The amplified and sequenced fragment was cloned via three-fragment ligation into vector pRRL.PPT.PGK.newMCS.EBFP2.i2.Zeo.PRE using the *Xho*I and *Nhe*I sites between the cPPT and the hPGK promoter sequences. The human codon-usage optimized ORF second generation CAR containing scFv (14.G2a), HCH_2_CH_3_ hinge, CD28, CD137 (4-1BB), and CD3ζ domains [25] was flanked by the restriction enzymes *Age*I and *Sal*I were cloned into the lentiviral “all-in-one” SIN vector driven by an hPGK promoter. The restriction sites *Mlu*I and *EcoR*I, or *Afe*I and *EcoR*I, respectively, were inserted to exchange the iGOI within the “all-in-one” vector construct (Figure 2A). To generate the lentiviral “all-in-one” SIN vector driven by 5’ CMV promoter, the fusion of the inducible element driven by NFAT, the constitutive CAR, and lentiviral backbone was obtained using *Not*I, *Age*I, and *Sca*I restriction sites. Constructs were validated by sequencing (Microsynth SeqLab, Germany). Cloning details and sequences are available on request.

Lentiviral particles were generated as described previously [51]. In brief, 5 × 10^6^ 293T cells were seeded. The transfection was performed using the calcium phosphate method in the presence of 25 µM chloroquine with the following plasmids: lentiviral vector plasmid (10 µg), pcDNA3.HIV-1.GP.4 × CTE (lentiviral gag/pol) (12 µg) [52], pRSV-Rev (5 µg; kindly provided by T. Hope, Northwestern University Chicago, IL), and VSVg-encoding pMD.G (1.5 µg) [53]. For better standardization, pcDNA3.HIV-1.GP.4×CTE, pRSV-Rev and pMD.G were produced and purified by PlasmidFactory (Bielefeld, Germany). Supernatants were harvested 36 h and 48 h after transfection and concentrated via ultracentrifugation at 4 °C and 13,238× *g* or 82,740× *g* for 16 h or 2 h, respectively.

Titration of viral supernatants was performed in HT1080 fibroblasts via spinoculation-mediated transduction (4.4.). Transduction efficiency was determined three days post transduction via flow cytometric staining of G_D2_CAR expression. Functional viral vector titers were calculated from samples with G_D2_CAR expression percentages of ≤30% to avoid cells with multiple integrations [54].

### 4.4. Transduction of Cell Lines and Primary T Cells

For spinoculation-mediated transduction of HT1080 fibroblasts, 1 × 10^5^ cells were seeded in a 24-well plate. Supernatants containing viral particles and 4 µg/mL protamine sulfate (Sigma-Aldrich) were added and cells were centrifuged (1 h, 800× *g*, 37 °C). The NFAT-GFP hybridoma cell line was transduced identically. Primary human T cells were isolated by magnetic activated cell sorting (MACS) from human peripheral blood mononuclear cells (PBMCs) previously isolated via density centrifugation. Subsequently, isolated T cells were activated for 48 h prior to transduction using T Cell TransAct^TM^ (αCD3/CD28 beads, Miltenyi) in complete RPMI1640 medium supplemented with 100 U/mL human IL-2. After activation, human T cells were transduced with the lentiviral constructs using Retronectin (Takara, Kusatsu, Japan). In brief, 48-well plates were coated with Retronectin (137 µL of 24 mg/mL in PBS) overnight, blocked with sterile-filtered PBS containing 2% BSA for 30 min, and washed with Hank’s Balanced Salt Solution (HBSS)/HEPES. Wells were loaded with lentiviral supernatants at the desired multiplicity of infection (MOI 3 or 10) by centrifugation for 30 min, 400× *g*, 4 °C, followed by adding 2.5 × 10^5^ T cells per well [55]. The transduction efficiency was analyzed 6–7 days post transduction by flow cytometry after staining the scFv with the Ganglidiomab-phycoerythrin (PE) mAb (kindly provided by H. Lode and N. Siebert and PE-conjugated) [56,57]. For some analyses, the transduced T cells were isolated via FACS-based cell sorting. This is indicated in the respective figure legends. For some experiments (Figure 6, Figure 7 and Figure S4), T cells were activated for 24 h with αCD3/CD28 beads (Miltenyi) and transduced by centrifugation for 45 min, 800× *g*, 32 °C with lentiviral constructs using polybrene (5 µg/mL) (Merck Millipore, Burlington, VT, USA) in complete TexMACS medium.

### 4.5. Hybridoma Co-Culture Assay

The NFAT-GFP hybridoma cell line (kindly provided by Ludger Klein (TU Munich, city, Germany)) encodes an EGFP under the control of an NFAT-dependent IL-2 promoter [58]. All hybridoma cultures and hybridoma-related assays were performed in DMEM supplemented with 10% FBS and 100 U/mL penicillin and 100 μg/mL streptomycin. The hybridoma cells were transduced with the lentiviral G_D2_CAR constructs by spinoculation (800× *g*, 1 h, 37 °C) in the presence of protamine sulfate. Transduction efficiencies were analyzed via flow cytometric staining 48 h post transduction. For functional tests, HT1080 and HT1080 GD2 cells were used as target cells. The co-culture assay was performed for 20 h with G_D2_CAR-transduced hybridoma cells with a 4:1 effector/target ratio (E/T) and analyzed via flow cytometric staining.

### 4.6. Flow Cytometric Analysis

Flow cytometry surface staining of T cell phenotype and activation markers were performed after co-culture experiments using the following anti-human antibodies CD3 (OKT3, eBioscience, San Diego, CA, USA), CD3 (OKT3, SK7), CD4 (RPA-T4), CD8 (SK1), CD69 (FN50), CD137 (4B4-1), CD25 (BC96), and CD154 (24-31) (all BioLegend, San Diego, CA, USA). GD2 expression on target cells was analyzed via an anti-human Ganglioside GD2 antibody (BioLegend).

### 4.7. Cytotoxicity Assay 

Cell-mediated cytotoxicity of primary T cells against GD2-expressing target cells (HT1080 GD2, SH-SY5Y neuroblastoma cell line) was determined via measuring the release of lactate dehydrogenase (LDH) with the Pierce^TM^ LDH Cytotoxicity Assay Kit (Thermo Scientific, Bremen, Germany) using co-culture supernatants according to the manufacturer’s instructions. Briefly, the indicated E/T ratio and appropriate controls (volume controls and maximum controls according to manufacturer’s protocol) were seeded in a 96-well plate. After 48 h of co-culture, 50 µL of the cell-free co-culture supernatants was transferred to another 96-well plate and incubated with 50 µL provided reaction mix for 30 min at room temperature, and then it was stopped by adding the provided stop solution. To determine LDH activity, absorbance at 490 nm and 680 nm was measured (SPECTRAmax 340PC, Molecular DEVICES, Software SOFTmax PRO 4.0) and subtracted (490 nm–680 nm), respectively. The % cytotoxicity was calculated according to the manufacturer’s protocol.

### 4.8. Cytokine Analysis by ELISA and LEGENDplex

Cell culture supernatants from primary T cells cultured alone or after indicated time points of co-cultures with HT1080, HT1080 GD2, SH-SY5Y, or primary glioblastoma cells were collected and cytokine concentrations were determined using the Human IL-12 (p70) ELISA MAX^TM^ Deluxe Set (BioLegend) and the Human Total IL-18 DuoSet^®^ ELISA Kit (R&D Systems, Minneapolis, MN, USA) according to the manufacturer’s instructions. Absorbance was measured with a SPECTRAmax 340PC plate reader (Molecular DEVICES, Biberach an der Riss, Germany, Software SOFTmax PRO 4.0). In addition, supernatants from in vitro co-cultures were analyzed using a customized LEGENDplex™ Multi-Analyte Flow Assay (BioLegend), which allowed for the simultaneous detection of human IL2, IL10, IL12 (p70), IL18, granzyme B, IFNγ, perforin, and TNFα. The assay was performed according to the manufacturer’s instructions. Briefly, undiluted samples and standards were incubated with antibody-coated premixed beads overnight at 4 °C followed by incubation with detection antibody for 1 h at room temperature and streptavidin-PE for 30 min at room temperature. Samples were acquired on a BD FACSCanto™ Flow Cytometer (BD Biosciences, Heidelberg, Germany) and analyzed with the LEGENDplex v8.0 Software (BioLegend).

### 4.9. Cell Migration Assay 

To test the chemoattractive potential of supernatants that were derived from co-culture experiments of primary human T cells transduced with the indicated lentiviral “all-in-one” constructs and GD2^+^ target cells, the migration assay with MONO-MAC-6 monocytes (MM6 cells) [59] was performed using a Boyden chamber (NeuroProbe, Gaithersburg, MD, USA) [60]. In brief, a 96-well plate (Sarstedt, flat bottom) was first filled with 300 µL/Agar per well. Then, 110 µL supernatant of co-culture experiments (different concentrations of the CC-chemokine ligand CCL2 were used as positive controls) were added per well and the plate was placed in the Boyden chamber using a spacer. Afterward, a polycarbonate membrane (8 µm pore size, NeuroProbe) was placed over the plate and the chamber was closed. Subsequently, 200 µL MM6 monocytes (5 × 10^5^ cells/well) were added to the upper chamber. The entire chamber was incubated for 4 h at 37 °C and 5% CO_2_. The membrane was removed and cells attached to the top of the membrane were removed with a scraper. Migrated cells attached to the lower side and inside the membrane were analyzed by fixing and staining the membrane with methanol for 10 min (Roth, Karlsruhe, Germany) and Giemsa for 1 h, respectively. The cell number was calculated using an Olympus IX71 microscope.

### 4.10. Microscopy

Brightfield images and NFAT-induced EGFP expression in primary T cells after co-cultures with patient-derived glioblastoma spheroids were analyzed and performed using an AxioObserver microscope (ZEISS, Jena, Germany) and AxioVision Software Rel. 4.8. Scale bars are indicated in each figure.

### 4.11. Statistical Analysis

Statistical analyses were performed with the help of GraphPad Prism version 6.0 (GraphPad software, San Diego, CA, USA). One-way ANOVA and two-way ANOVA tests with Tukey’s or Bonferroni’s multiple comparison test and unpaired two-tailed *t*-test; *** *p* ≤ 0.001; ** *p* ≤ 0.01; * *p* ≤ 0.05; ns > 0.05 were used. Analyses and *p*-values are also specified in the figure legends.

## 5. Conclusions

In conclusion, the described “all-in-one” vector concept with its modular design and its combination of constitutive CAR expression and inducible NFAT-driven transgene expression provides a useful vector concept for targeted gene transfer for adoptive gene therapies, especially regarding easy vector transfer and clinical application. Thus, with the “all-in-one” vector design, improved vector titer capacities, and superior inducibility, our system will be useful for the scientific community as it is suitable for various manufacturing processes for therapeutic purposes. Moreover, the system will be applicable to any other settings in which expression of one constitutive and one inducible gene cassette is desirable.

## Supplementary Materials

The following supplementary figures are available online at https://www.mdpi.com/2072-6694/12/2/375/s1, [Note to journal staff: Please add as correct], Figure S1. Co-culture of transduced T cells with GD2^-^ target cells led to a moderate EGFP upregulation, Figure S2. Human IL12 and human IL18 secretion after stimulation with GD2^-^ target cells, Figure S3. Inducible NFATmIL2-driven human IL12 and human IL18 secretion after CAR-specific stimulation, Figure S4. Cytokine analysis after G_D2_CAR-specific activation in human T cells transduced with “all-in-one” vector constructs.
