# Supplementary material for: Design and Characterization of an “All-in-One” Lentiviral Vector System Combining Constitutive Anti-GD2 CAR Expression and Inducible Cytokines
Source: Cancers (Basel). 2020 Feb 6;12(2):375. doi: 10.3390/cancers12020375 (PMC7072617; doi:10.3390/cancers12020375)
Supplement: Supplementary file 1 [file cancers-12-00375-s001.pdf]

Supplementary Figures: Design and Characterization of an "All-in-One" Lentiviral Vector System Combining Constitutive Anti-GD2 CAR Expression and Inducible Cytokines

Katharina Zimmermann, Johannes Kuehle, Anna Christina Dragon, Melanie Galla, Christina Kloth, Loreen Sophie Rudek, I. Erol Sandalcioglu, Belal Neyazi, Thomas Moritz, Johann Meyer, Claudia Rossig, Bianca Altvater, Britta Eiz-Vesper, Michael Alexander Morgan, Hinrich Abken and Axel Schambach

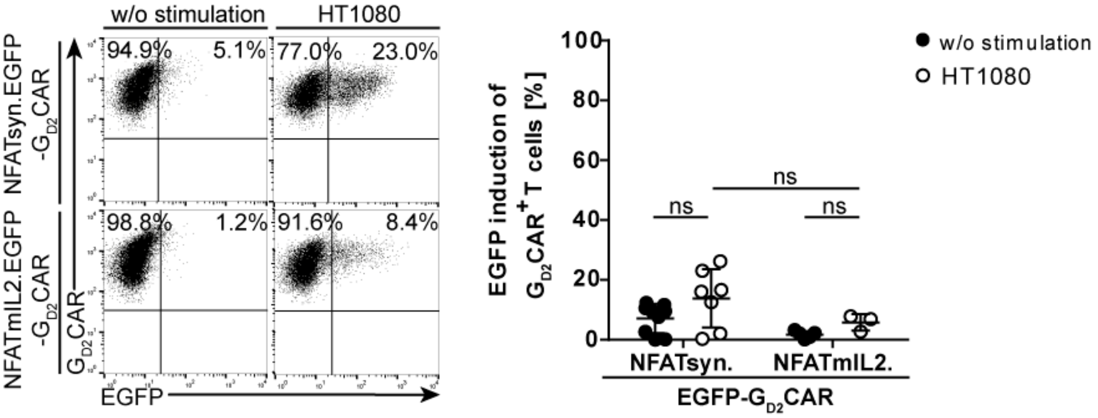

Figure S1. Co-culture of transduced T cells with GD2$^-$ target cells led to a moderate EGFP upregulation. Comparison of inducible promoters, namely NFATsyn and NFATmIL2. Primary human T cells were transduced with "all-in-one" EGFP constructs (NFATsyn.EGFP-GD2CAR or NFATmIL2.EGFP-GD2CAR), sorted and co-cultured with GD2$^-$ target cells. GD2CAR-specific activation with HT1080 cells (GD2-negative) results in a moderate NFAT promoter-regulated EGFP expression similarly to the control without any target cells. Representative flow cytometric analysis of NFAT-driven EGFP expression in transduced T cells after 24 h co-culture with a 6:1 effector:target (E:T) ratio. Data shown are from the same experiment displayed in Figure 3. The graph summarizes multiple experiments of NFAT promoter-driven EGFP expression in "all-in-one" construct-positive (GD2CAR$^+$) T cells post stimulation with HT1080 target cells. Shown are mean values ± SD; symbols indicate individual co-culture experiments with individual donors; n = 3–8. Indicated statistical significance was determined by one-way ANOVA with Tukey's multiple comparison test; ns > 0.05).

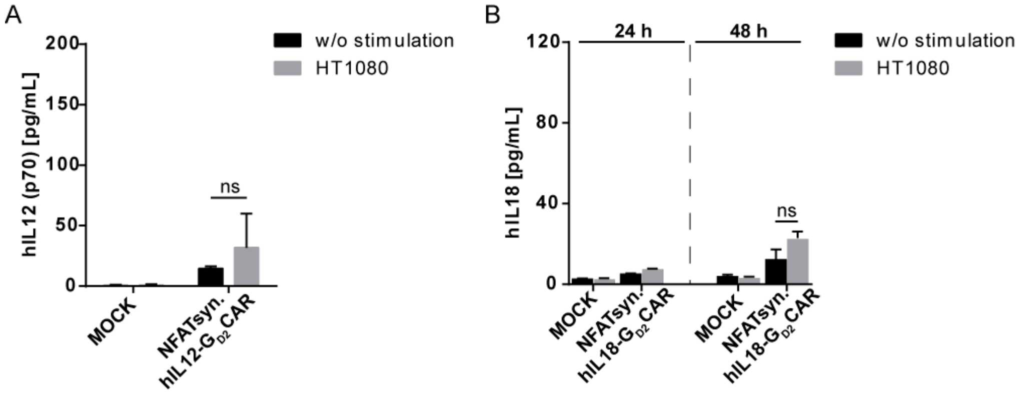

Figure S2. Human IL12 and human IL18 secretion after stimulation with GD2$^-$ target cells. (**A**) Human IL12 expression and (**B**) human IL18 expression in cell culture supernatants. Primary T cells were transduced with

"all-in-one" NFATsyn.hIL12-G_{D2}CAR and NFATsyn.hIL18-G_{D2}CAR constructs and co-cultured with $GD2^-$ target cells (HT1080) for 24 h (and 48 h) or without any cells. Inducible hIL12 or hIL18 secretion was determined via ELISA. ELISAs were performed in triplicates in two (hIL18) or three (hIL12) independent experiments; unsorted cells; 10:1 effector:target (E:T) ratio. Shown are mean values ± SD. Indicated significance was determined by unpaired two-tailed *t*-test; (ns > 0.05).

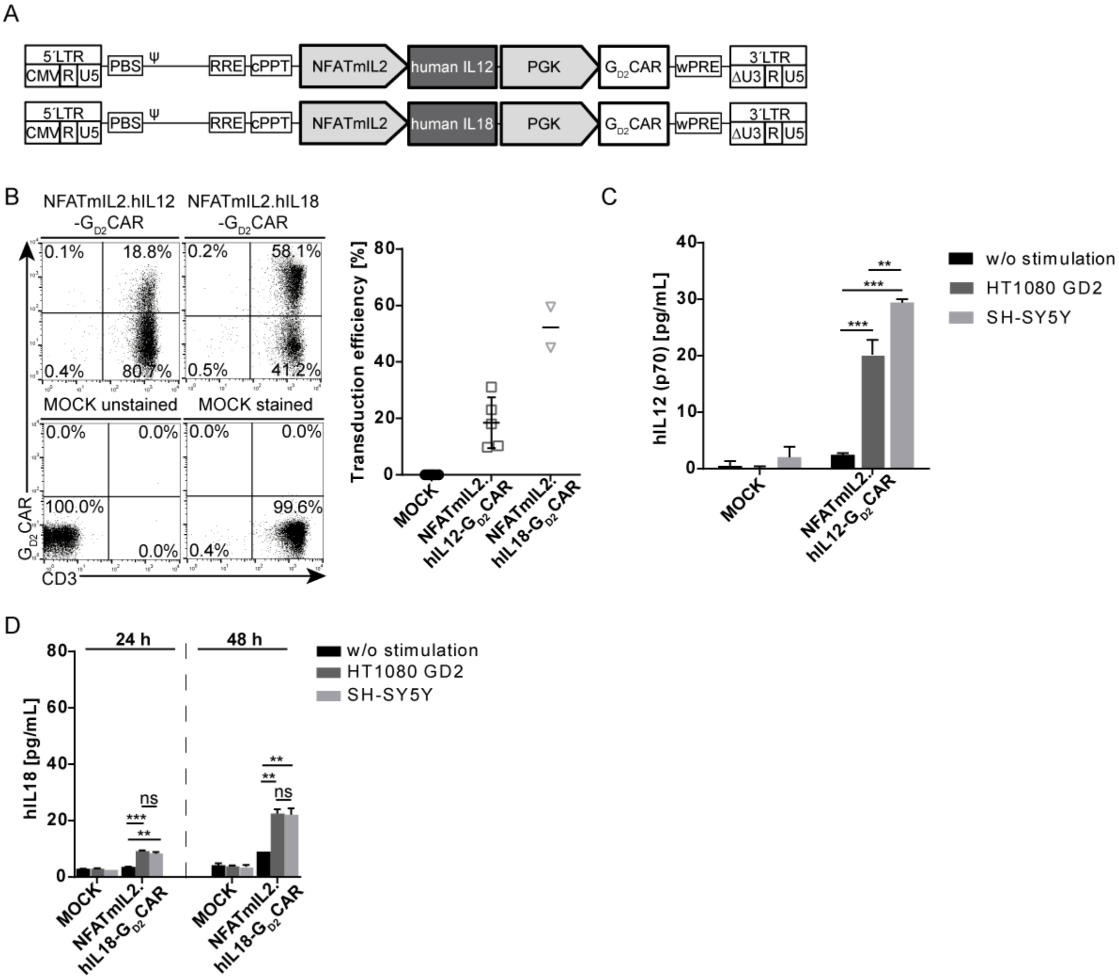

Figure S3. Inducible NFATmIL2-driven human IL12 and human IL18 secretion after CAR-specific stimulation. (**A**) Schematic design of the "all-in-one" NFATmIL2.hIL12-G_{D2}CAR and NFATmIL2.hIL18-G_{D2}CAR constructs. Indicated is the inducible promoter (NFATmIL2). (**B**) Representative flow cytometric analysis of the "all-in-one" NFATmIL2.hIL12-G_{D2}CAR and NFATmIL2.hIL18-G_{D2}CAR constructs in transduced primary human $CD3^+$ T cells (MOI 10). Transduction efficiency was determined six days post transduction via a G_{D2}CAR-specific antibody that recognizes the scFv region. Graph summarizes transduction efficiencies of the "all-in-one" NFATmIL2.hIL12-G_{D2}CAR and NFATmIL2.hIL18-G_{D2}CAR constructs in primary human T cells. Shown are mean values ± SD (symbols indicate individual donors; n = 2–5). (**C**) Human IL12 expression and (**D**) human IL18 expression in cell culture supernatants. Primary human T cells were transduced with "all-in-one" NFATmIL2.hIL12-G_{D2}CAR and NFATmIL2.hIL18-G_{D2}CAR constructs and co-cultured with $GD2^+$ target cells for 24 h (or 48 h) in a 10:1 effector:target (E:T) ratio. Inducible IL12 or IL18 secretion was determined via ELISA. ELISAs were performed in triplicates in two independent experiments. Shown are mean values ± SD. Indicated significance was determined by one-way ANOVA with Bonferroni´s multiple comparison test; *** $p \leq 0.001$; ** $p \leq 0.01$; * $p \leq 0.05$; ns > 0.05.

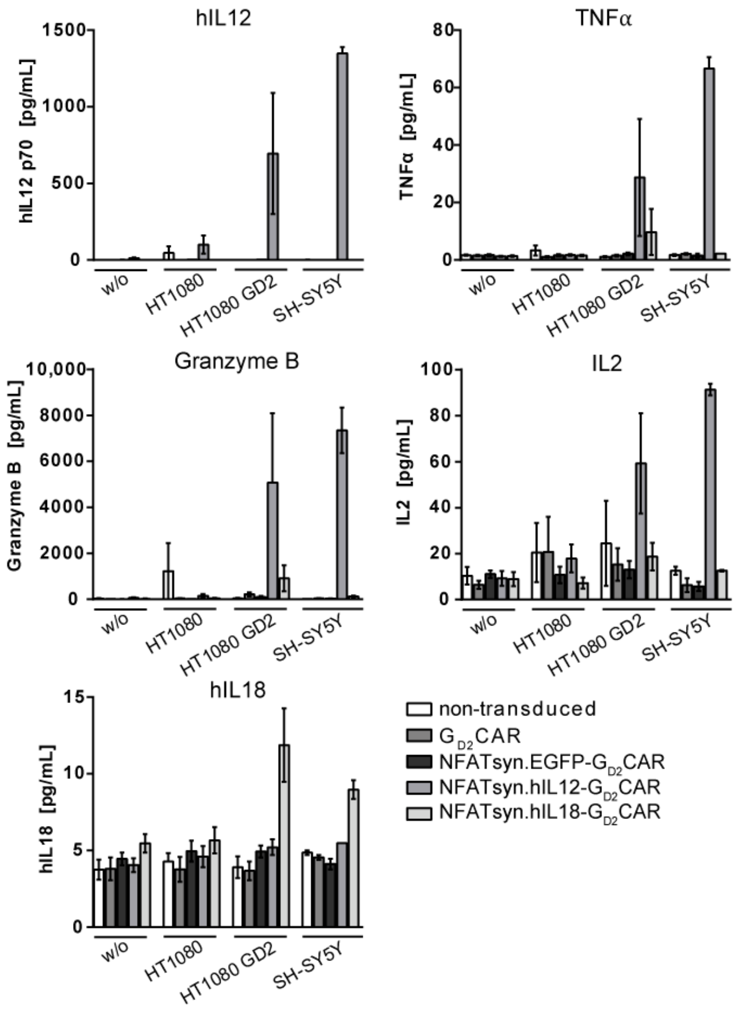

Figure S4. Cytokine analysis after GD2CAR-specific activation in human T cells transduced with "all-in-one" vector constructs. Cytokine concentrations in co-culture supernatant were determined after 48 h co-culture experiments of transduced primary T cells stimulated with indicated target cells in a 6:1 effector:target (E:T) ratio using a customized LEDGENDplex™ Multi-Analyte Flow Assay (BioLegend), which allowed simultaneous detection of human IL12, TNFα, IFNγ, granzyme B, IL2, perforin and human IL18. Shown are mean values ± SD (symbols indicate individual donors; *n* = 2–5).

© 2020 by the authors. Licensee MDPI, Basel, Switzerland. This article is an open access article distributed under the terms and conditions of the Creative Commons Attribution (CC BY) license (http://creativecommons.org/licenses/by/4.0/).
